# Perspectives on artificial intelligence in healthcare from a Patient and Public Involvement Panel in Japan: an exploratory study

**DOI:** 10.3389/fdgth.2023.1229308

**Published:** 2023-09-13

**Authors:** Amelia Katirai, Beverley Anne Yamamoto, Atsushi Kogetsu, Kazuto Kato

**Affiliations:** ^1^ Research Center on Ethical, Legal, and Social Issues, Osaka University, Suita, Japan; ^2^ Graduate School of Human Sciences and Headquarters, Osaka University, Suita, Japan; ^3^ Department of Biomedical Ethics and Public Policy, Graduate School of Medicine, Osaka University, Suita, Japan

**Keywords:** artificial intelligence, healthcare, patient and public involvement, Patient and Public Involvement Panel, community participation

## Abstract

Patients and members of the public are the end users of healthcare, but little is known about their views on the use of artificial intelligence (AI) in healthcare, particularly in the Japanese context. This paper reports on an exploratory two-part workshop conducted with members of a Patient and Public Involvement Panel in Japan, which was designed to identify their expectations and concerns about the use of AI in healthcare broadly. 55 expectations and 52 concerns were elicited from workshop participants, who were then asked to cluster and title these expectations and concerns. Thematic content analysis was used to identify 12 major themes from this data. Participants had notable expectations around improved hospital administration, improved quality of care and patient experience, and positive changes in roles and relationships, and reductions in costs and disparities. These were counterbalanced by concerns about problematic changes to healthcare and a potential loss of autonomy, as well as risks around accountability and data management, and the possible emergence of new disparities. The findings reflect participants' expectations for AI as a possible solution for long-standing issues in healthcare, though their overall balanced view of AI mirrors findings reported in other contexts. Thus, this paper offers initial, novel insights into perspectives on AI in healthcare from the Japanese context. Moreover, the findings are used to argue for the importance of involving patient and public stakeholders in deliberation on AI in healthcare.

## 1. Introduction

Public and private investments in the development of artificial intelligence (AI) technologies for healthcare are being rapidly expanded. AI for healthcare as currently conceived involves the ability to process and learn from massive amounts of data, and includes machine and deep learning, expert systems, natural language processing, healthcare informatics, and cloud computing, as well as applications in robotics (1). Despite these investments, there is a growing recognition that little is known about the views of the end-users of these systems, including patients, members of the public, and healthcare professionals (HCPs) (2). Given the particular challenges of implementing AI in healthcare, including the sensitivity of the data involved, there have been calls for increased stakeholder—including patients and the public—involvement in the development of AI for healthcare (1).

There is a growing body of literature seeking to fill this gap, which has been captured by a recent review of the field by Young et al. (3), who found that in the 23 included studies in their study, patients and members of the public generally expressed positive views on the use of AI, but also “had many reservations and preferred human supervision”. Two recent studies, by Richardson et al. (2) and Musbahi et al. (4) sought to elicit patient and public perspectives on the application of AI in healthcare more broadly, and similarly found some ambivalence towards AI, with recognition of both its potential benefits and its risks.

However, despite groundbreaking efforts such as those by Muto and Inoue (5) and Kodera et al. (6), little research to date has examined patient and public opinions on AI in healthcare in the Japanese context. There has also been little dissemination of these results more broadly, as a recent review by Young et al. (3) of English-language literature reported no studies from the Japanese context. Yet, insights from the Japanese context can provide a counter-balance to an overly Western-dominated discourse on AI (7).

As Ishii et al. (8) indicate, Japan is a key case study through which to explore the opportunities and issues of AI in healthcare as it has “a technologically savvy populace, well-developed healthcare system founded on universal coverage, and pre-existing academic, government, and industrial collaborative alignments”. Japan has been actively investing in the development of AI for healthcare purposes, while the regulatory environment is being adjusted to facilitate the agglomeration and use of personal data on health (8). These advances are being carried out in part through a Cross-Ministerial Strategic Innovation Promotion Program implemented by the national government, which includes a target for the creation of ten “AI Hospitals”, receiving funding to integrate AI into healthcare practice (9, 10). Osaka University Hospital is one of the five hospitals where this process is currently being accelerated (11).

Patient and Public Involvement (PPI) in healthcare is increasingly relied on to ensure that advances in healthcare best meet the needs of their end-users (12). A collaborative research project entitled “Ensuring the benefits of AI in healthcare for all: Designing a Sustainable Platform for Public and Professional Stakeholder Engagement” (the AIDE Project) and jointly funded by JST-RISTEX in Japan and the UKRI in the UK is being carried out between teams at Osaka University in Japan and the University of Oxford in the UK. Now in its fourth and final year, the aim of the AIDE Project is to advance stakeholder engagement for AI in healthcare. For this reason, our work on the AIDE Project has taken a novel approach, guided by Patient and Public Involvement Panels (PPIPs) established in both Osaka and Oxford, to advise the research teams and provide insights on AI in healthcare from a patient and public perspective. It is noteworthy that despite initial efforts by the Japan Agency for Medical Research and Development to increase awareness of the importance of PPI (13), PPIPs remain few in number in the Japanese context, and there is a lack of infrastructure and structured support for their establishment.

We conducted a two-part, exploratory workshop with members of the Osaka PPIP, to understand participants' expectations and concerns about AI in healthcare. In this paper, we report the results of an analysis of the workshop outcomes, with the aim of providing a snapshot of patient and public perceptions of AI in healthcare in the Japanese context, and identify areas for future attention.

## 2. Methods

At the time of the workshop, the Osaka AIDE PPIP was made up of 11 members, with a balanced representation of patients, caregivers, and members of the public. Panel members ranged in age from their 20s to their 70s, with a balance of participants identifying as male and female. All participants were Japanese, and although no objective measure was taken of participant knowledge about AI, no participants declared having particular expertise in the field of AI.

Following an initial orientation session for the PPIP in early 2021 in which a researcher from the Osaka University AI Hospital Project was invited to speak to the Panel about advances in AI for healthcare, a two-part workshop was conducted with PPIP members to elicit their expectations and concerns in relation to AI. Ethics approval was received through the Osaka University Graduate School of Medicine [Number 20083(T1)-3], and informed consent was received from all PPIP members for the use of this data for research. Feedback was given to PPIP members following the workshop on the themes which were identified from the data.

The workshop sessions were held on February 25 and April 21, 2021. PPIP members were divided into three groups and asked to freely record their expectations and concerns about the use of AI in healthcare, without input from the research team. A Japanese method was selected for the workshops: the “Science Café” participatory workshop approach, proposed by Nakagawa and Yagi (14), was adapted so as to enable to the workshop to be conducted online due to the Covid-19 pandemic. Nakajima et al. (15) propose the use of the online platform Apisnote (16) to facilitate idea-sharing in online workshops. Aligned with this approach, in the first workshop, PPIP members were first given time for individual reflection, following which they were asked to post their ideas (items) to Apisnote. Following this, each member was given time to present on their items. In the second session, each group was asked to review, cluster, and title the expectations and concerns which they had identified in the previous session. In this process, participants were asked to keep a distinction between those items that were expectations and those that were concerns through color-coding.

The results were translated into English, with the aim in the translation process being to remain as close as possible to the original phrasing and nuance of the Japanese. As each group's titles were unique but contained overlap, thematic content analysis was used to synthesize the items across groups and to identify overarching trends in the data. The titles created by group members—rather than the items themselves—were coded, to better reflect the clustering work by PPIP members. The data was coded through an inductive process, and codes were then merged to form overarching themes, which were then reapplied to the dataset (See Appendix for further details.) The results of this analysis are reported below.

## 3. Results

Across the three groups, a total of 107 items were identified. Of this total, 55 (51%) were expectations, while 52 (49%) were concerns. Six themes reflected expectations, while six reflected concerns. This suggests a perception of AI in healthcare among the PPIP members that is balanced overall. Through the analytic process, these items were clustered and categorized into 12 themes by AmK with review by BY.

Below, the overarching themes and the participant-generated titles within each theme will be discussed. The extracts included below are participant postings, translated by AmK with review from all co-authors.

### 3.1. Expectations

There were six themes reflecting expectations for AI in healthcare. These are reported in Table 1 below, where the first item in each row is the researcher-generated overarching theme and is followed by the participant-generated bulleted items.

**Table 1:** Participant expectations for AI in healthcare.

Themes (heading) and participant-generated titles (bulleted)	Number of items	% of total expectations	% of total items generated
Improved hospital administration			
• Securing human resources to work as HCPs • Securing healthcare resources • Improving work efficiency in hospitals • Increased profitability of hospital management • Improving hospital management	14	25%	13%
Improved quality of care			
• Improvement in technology for treatment • Smoother diagnosis and treatment • Early diagnosis	11	20%	10%
Positive changes in roles and relationships			
• AI as an intermediary between experts and the public • Friendly relationships with AI (outside of medicine) • The importance of both AI and HCPs • Closer relationships to experts • Deeper communication and helpfulness • Excitement about the future • Personalized avatar doctors as desirable	9	16%	8%
Cost reductions			
• Leading to cost reduction • Reducing the financial burden on patients	8	15%	7%
Better patient experience			
• Reduction in anxiety and stress • Increased convenience (accessibility) for patients • Stimulates curiosity	7	13%	6%
Reducing disparities			
• Reducing medical disparities between hospitals • Elimination of regional disparities • Parity in the quality of medical care	6	11%	6%
Total	**55**		

Expected improvements in hospital administration was the most prominent theme across the results and made up 13 percent of the total items. PPIP members expressed expectations that AI would help to offset the lack of human and other resources needed for healthcare. This included the expectation that AI would enable the provision of healthcare even in remote areas and other places where there may be shortages of doctors. Moreover, it was expected that hospitals would function more efficiently in areas ranging from the management of medicines and prescriptions to clinical trials, leading to increased profitability. HCPs would benefit from a reduction in burden and in long working hours, as rote work would be reduced. This would allow them to devote time to fulfilling their true role as healthcare professionals (Extract 1).

#### Extract 1 (Group 2)

*The possibility that healthcare professionals will be able to concentrate on the work that they should be able to focus on*

The next-largest theme, comprising 10 percent of all items, was improved quality of care. This dealt with the possibility that AI implementation would lead to improvements in diagnosis and treatment. Patients would have increased access to healthcare-related information, and access to remote care would be expanded (Extract 2).

#### Extract 2 (Group 2)

*Possibility of clinical examinations and treatment from home for people in remote areas, the elderly, and people with disabilities*

This tied in with a further theme, which was the expectation that there would be positive changes to roles and relationships. AI was expected to facilitate better communication in clinical settings, and overall, there was the expectation that AI would become a familiar entity in patients' lives (Extract 3), with hopes for personalized interactions.

#### Extract 3 (Group 1)

*Excited about the future of healthcare because AI will be something the children will be familiar with going forward*

Furthermore, and closely linked with the first theme, PPIP members expected that AI would improve the diagnostic and treatment processes, which would lead to a reduction in the financial burden on patients through a broad range of knock-on effects, including the increased use of generic drugs and a reduction in medical expenses for patients, who generally have to cover 30% of costs under the universal healthcare care system. Participants expected that the use of AI would also facilitate outpatient triage and allow for increased data portability as patients would be able to access and search within their own medical information, which was seen to be a part of cost reduction criteria (Extract 4).

#### Extract 4 (Group 3)

*It will become easier to accumulate and search (personal) information*

PPIP members expected that AI would facilitate a better patient experience through a reduction in the anxiety and stress involved in hospital visits, and increased convenience for patients. One participant referred to the exhausting nature of hospital visits at present (Extract 5). There were hopes that AI would lighten the burden of hospital visits by simplifying administrative procedures. Moreover, these procedures would be more accessible to patients who did not speak Japanese, who could benefit from more user-friendly systems. Some participants expressed a personal interest in AI, and saw it as something exciting and of interest, which they said would be appreciated by those who enjoy interacting with new technologies.

#### Extract 5 (Group 3)

*(From the perspective of patients) I expect that procedures at the hospital will be simplified and that waiting times will be shortened. I also expect that the system will be user-friendly for people with disabilities and non-Japanese speakers, who experience barriers to access to information. I hope that hospital visits will no longer exhaust patients and lead to a breakdown in their health*

PPIP members also expected that AI would reduce disparities in the quality of care experienced by patients in different regions (i.e., remote or rural areas), or at different types of hospitals (i.e., smaller clinics and larger research hospitals), making medical treatment more accessible to patients regardless of location. At the same time, they expected that remote care would be expanded, thus reducing the physical burden of travel to receive care, and reducing disparities by increasing access to quality care for those living in remote areas. This would reduce the concentration of patients visiting large hospitals (Extract 6).

#### Extract 6 (Group 2)

*Standardization of the level of healthcare, elimination of the concentration of patients at large hospitals*

### 3.2. Concerns

Although PPIP members noted a variety of expectations for AI in healthcare, they also had concerns about its implementation. There were six themes which reflected concerns about AI in healthcare, as in Table 2.

**Table 2:** Participant concerns about AI in healthcare.

Themes (heading) and participant-generated titles (bulleted)	Number of items	% of total concerns	% of total items generated
Concerns about changes to healthcare			
• Concerns related to the true nature of healthcare • Changes in the structure of the healthcare system • Deterioration of doctors’ diagnostic ability due to reliance on AI • What will happen in an age where AI is seen to be absolute? • What will happen in an age where AI surpasses doctors? (Both to patients and doctors)	13	25%	12%
Limitations and loss of autonomy			
• Can AI understand the hearts/minds of patients/people? • Anxiety caused by what AI is lacking • Loss of treatment options and possibilities • Can AI be used effectively? • Negative perceptions of AI	11	21%	10%
Technical issues and accountability			
• Ensuring the quality and accuracy of information • Program update and backup issues • Misdiagnoses, accidents, and accountability issues	9	17%	8%
Emerging disparities			
• Disparities between people comfortable with new technologies and those who are not • Issues around disparities • Problems with literacy in relation to personal information/use of devices • Differences in implementation depending on the region, hospital, and doctor • Need to make the purpose of AI development clear	7	13%	7%
Data management issues			
• Concerns about the system • Risk of leaks of personal information • Problems with the handling of personal information	7	13%	7%
Costs of implementation			
• The costs of and time needed for implementation • Mistrust of the system	5	10%	5%
Total	**52**		

The second largest overall theme identified in the analysis was that of concerns about changes in healthcare, which made up 12% of total items. One set of concerns was that AI may move healthcare away from its “true nature”, or how participants felt it “should” be, leading to problems in clinical relationships and in the structure of healthcare in the future. This related to concerns that doctors' diagnostic skills may be surpassed by AI, and their roles as decisionmakers would be undermined. PPIP members indicated concern about a possible negative impact on the relationship between doctors and patients, if doctors were to become over-reliant on AI, and if decisions from black-boxed algorithms were to be accepted as “absolutes”, with little room for reconsideration or second opinions (Extract 7). Moreover, contrary to the expectations discussed above, if these systems were to be made available at large hospitals, there could be an increased over-concentration of patients at large hospitals seeking out AI-powered healthcare, even if they could otherwise be treated elsewhere.

#### Extract 7 (Group 1)

*I think that in healthcare, the language of “absolutes” is avoided, but AI healthcare may come with such absolutes*

The second largest cluster of concerns was the perceived limitations of AI and potential loss of autonomy for both HCPs and patients. There was an implicit assumption that the introduction of AI into healthcare would require direct communication between patients and robots or other AI-powered entities, to which care would be delegated. This appeared, for example, in PPIP members' concerns that patients would struggle to fully express themselves in interaction with AI, or that they may experience psychological anxiety at not being able to meet “the real thing” (i.e., a human HCP; Extract 8).

#### Extract 8 (Group 1)

*Psychological anxiety due to no longer being able to meet the real thing*.

For this reason, although participants expected that AI could improve communication, as described above, there were also concerns that the technology would not have the capacity to understand patients' thoughts and feelings, and that this would impede communication and prevent them from freely expressing themselves. This tied into an overarching perception of AI as “cold”, as opposed to the implied warmer nature of human-centered healthcare. Furthermore, there were concerns that delegating care to AI-based systems would mean a loss of autonomy for patients in pursuing treatment options. Participants worried about being made to use new machines, or that they may be subject to medical decisions that they are not prepared for. One example given by a PPIP member was of being recommended surgery for which the patient is totally unprepared (Extract 9).

#### Extract 9 (Group 1)

*Things will advance suddenly in directions unanticipated by patients (Such as having surgery suddenly recommended without any preparation)*

Accuracy of the output from AI was another concern, and how legal and other issues related to accountability would be managed, such as if system errors or failures occurred which led to fatal outcomes. PPIP members also expressed concerns about whether bugs in the system would be fixed appropriately and questioned what would happen if online platforms failed due to natural or other disasters (Extract 10).

#### Extract 10 (Group 2)

*Issues of backups when online platforms are unavailable due to natural disasters, etc*.

Although, as described above, there were expectations that AI would reduce disparities, there were contradictory concerns that it would expand them at the levels of individuals, hospitals, and regions. At the individual level, participants worried that some people may not be comfortable with new technologies. They thought that not all hospitals may be able to implement new technologies equally, and that there may be regional disparities both in implementation and in the ability of doctors or hospitals to handle new technologies. One participant expressed concern about the risk for expanded disparities on a global scale (Extract 11).

#### Extract 11 (Group 1)

*For what purpose are we developing AI? If we have an awareness of humanity as a whole, if it is only available in particular environments, won't this only serve to exacerbate disparities?*

The final area of concern was around the possible costs of implementation and whether investments would bear fruit. There was unease about the possibility that the investment of time and money into developing AI may not pay off. Participants observed that other major investments in system reform paid for with taxpayer money had come to nothing, and so there was a degree of skepticism about investments in AI (Extract 12).

#### Extract 12 (Group 3)

*Many systems created with taxpayer money cannot be used—will that not happen?*

## 4. Discussion

Overall, this exploratory workshop with the AIDE Project PPIP highlighted the meaningful input patients and members of the public can provide on AI for healthcare in the Japanese context. This is notable given that PPI in Japan continues to be underdeveloped, and consultation with stakeholders on AI remains limited.

The results of this study reflect PPIP members' perceptions of issues in healthcare broadly, and their expectations for AI as a possible remedy for them, as exemplified through the key question raised in Group 1 above, of what the purpose behind AI development truly is (Extract 11).These issues as reflected in participants' postings include the limited availability of human and financial resources for healthcare, the need for greater efficiency and accuracy, issues in patient experience, and disparities between hospitals and regions within Japan. The expectations of participants that AI will improve healthcare align with those expressed in reports promoting its implementation in healthcare [e.g., (11, 17)]. Participants expected there would be the potential for better hospital administration, an improved quality of care and patient experience, positive changes in roles and relationships, and a reduction in disparities. Thus, it is noteworthy that the PPIP held a balanced perception of AI, with a nearly-even split between expectations and concerns in the items elicited. This mirrors the ambivalent approach of patients and members of the public toward the implementation of AI in healthcare identified in other contexts [e.g., (3)].

Recent research such as by Richardson et al. (2) and Musbahi et al. (4) has sought to identify patient and public expectations and concerns about AI in healthcare broadly and can offer a source for comparative cross-cultural insights. The themes in this study echo those found in Richardson et al.'s (2) study in which participants were “generally enthusiastic”, about AI, but held concerns about the potential impact of AI on the autonomy of patients, concerns about rising healthcare costs, data quality, and concerns about security. These themes overlap with the themes identified in our study, though data quality was not articulated as a prominent concern by our PPIP. Similarly, many of the expectations and concerns emerging from Musbahi et al.'s study were also reflected in this study, including expectations for faster diagnosis, the possibility of AI-powered triage, a reduction in rote work, greater efficiency, AI as a helpful source of information, AI as an equalizer to reduce disparities, and AI as a cost-saver. There was also overlap in the concerns elicited, including about privacy and data management, issues around accountability, and the risk of deterioration in HCPs' skills (4)—also reported by Jutzi et al. (18).

The concern about the potential loss of the human touch in healthcare was a key theme here which has been reported in other settings, such as by Nelson et al. (19), Adams et al. (20), Yang et al. (21), and Young et al. (3). This perception that AI implementation may result in increased anxiety as due to the loss of the “emotional side” (4, 19, 20) of clinical relationships emphasizes the urgency of ensuring that human skill in healthcare is enhanced rather than replaced (22).

There were also some notable absences in the themes from this Japanese workshop given recent literature on the ethics of AI for healthcare. These included a lack of expressed concern around bias (23–25)—where disparities were raised, they were generally in relation to differences in the quality of healthcare between regions within Japan, and between different types of healthcare providers. There was no expression of concern about the insufficiency of current regulatory frameworks for AI in healthcare, the export of AI into other regions internationally, or of commercial involvement (8). Also missing was concern over sustainability issues across the life-course of AI (26). Further research is needed to determine to what extent patients and the public in Japan are aware of these issues around AI, and how to facilitate information-sharing about these risks. Moreover, it is noteworthy that participants themselves did not propose increased stakeholder involvement or engagement around AI in healthcare in this workshop, although some participant-generated items pointed to the introduction of AI itself as one way through which to increase patients' involvement in their own care.

A further aspect of the findings was the broad and future-oriented range of AI applications implied by the items elicited. Several of the items centered around possible applications of AI which are more advanced AI than the narrow applications available for real-world deployment. There was an implicit assumption that AI would replace clinicians, or that clinicians would be entirely reliant on AI. This contrasts with findings from studies by Yang et al. (27) and Jutzi et al. (18), where patients did not expect clinicians to be replaced by AI. It is also does not reflect the current reality in Japan, where approval of AI is limited, and only HCPs are permitted to make medical decisions (28). This future-orientation may reflect the challenges for stakeholders in understanding the entirety of AI systems and their applications and a need for greater information-sharing (29, 30).

### 4.1. Limitations and future directions

This study was intended as a small-scale, exploratory study spotlighting opinions of PPIP members on AI in healthcare. Though generalizability of the findings is limited, the qualitative orientation of this study and the small sample size ensured that the unique voices of each participant were well-reflected in the findings. Furthermore, this study utilized a Japanese method in eliciting stakeholder views on AI in healthcare—a novel contribution to the field. Bounds were not set around the types of AI under consideration to allow participants to situate the discussions around the technologies they found to be most of relevance or concern.

There is a need for the voices of otherwise marginalized or vulnerable stakeholders to be centered in deliberation on AI in healthcare (31). Both patients and caregivers were represented on the PPIP and offer perspectives on one aspect of potential vulnerability. Future research should actively seek out the perspectives of diverse individuals to investigate whether these themes remain consistent in a more diversified population. The AIDE Project research team is engaged in ongoing research with multiple stakeholder groups, with parallel but distinct involvement from a UK-based PPIP.

There is a growing consensus that consideration is urgently needed of the implications of AI for healthcare prior to its implementation. The meaningful involvement of stakeholders in these processes, including patients and members of the public is essential. This study has shown that patients and members of the public are keen to be engaged around AI in healthcare. It is crucial that they be given the opportunity to do so.

## Data Availability

The raw data supporting the conclusions of this article will be made available by the authors, without undue reservation.

## Appendix

The following is a description of the analytic process used for this study.

As the workshop was conducted online, participants were asked to post their comments to Apisnote directly. For this reason, the first analytic task was to put the data into a form through which it could be analyzed. Thus, it was manually copied into an Excel table with the following categories: the group number, whether participants had identified the posting as an expectation or a concern, the content of the posting, and the title for the cluster in which that posting was included. Certain groups also further clustered multiple titles into broad areas, and this was included in the table as needed. All items were translated into English, and both the English and Japanese were retained in the table.

There was a total of 48 titles (clusters) created by participants across the three groups. As noted above, there was overlap in these titles across groups, and for this reason, the titles were coded to find these commonalities. However, an analytic decision was made to code titles rather than the items (postings) themselves, to prioritize participants' own understandings and clustering of the items they generated.

An inductive process was used to code these titles, and codes were refined and readjusted multiple times until they best captured the data. Multiple codes were collapsed together to form overarching themes. Then, these themes were applied to both the titles and the items which participants had themselves grouped together under each title. This allowed for data to be extracted about the number of items under each theme, across the groups.

This process involved decision-making by the researchers about the appropriate thematic categorization of the titles. Although the data for this study was solely made up of participant postings, this decision-making was informed by the researchers' understandings gleaned from their experiences with participants in the workshop, in which the postings were discussed by participants. However, there were cases in which the participant-generated title (and thus the theme developed through coding) did not necessarily match all of the items under a particular participant-generated title. Given the decision to prioritize participant-generated clustering and titling of items, no items were moved from one cluster to another by the researchers, but rather were left as organized by participants, even when another categorization was conceivable.
